# Study of Protein Phosphatase 2A (PP2A) Activity in LPS-Induced Tolerance Using Fluorescence-Based and Immunoprecipitation-Aided Methodology

**DOI:** 10.3390/biom5031284

**Published:** 2015-06-29

**Authors:** Lei Sun, Adlai L. Pappy II, Tiffany T. Pham, Thomas P. Shanley

**Affiliations:** Division of Critical Care Medicine, Department of Pediatrics and Communicable Diseases, C.S Mott Children’s Hospital, University of Michigan Medical School, Ann Arbor, MI 48109, USA; E-Mails: leisun@med.umich.edu (L.S.); adlaipappyii@gmail.com (A.L.P.); tphammer@med.umich.edu (T.T.P.)

**Keywords:** PP2A, immune tolerance, phosphatase assay

## Abstract

Protein phosphatase 2A (PP2A) is one of the most abundant intracellular serine/threonine (Ser/Thr) phosphatases accounting for 1% of the total cellular protein content. PP2A is comprised of a heterodimeric core enzyme and a substrate-specific regulatory subunit. Potentially, at least seventy different compositions of PP2A exist because of variable regulatory subunit binding that accounts for various activity modulating numerous cell functions. Due to the constitutive phosphatase activity present inside cells, a sensitive assay is required to detect the changes of PP2A activity under various experimental conditions. We optimized a fluorescence assay (DIFMU assay) by combining it with prior anti-PP2A immunoprecipitation to quantify PP2A-specific phosphatase activity. It is also known that prior exposure to lipopolysaccharides (LPS) induces “immune tolerance” of the cells to subsequent stimulation. Herein we report that PP2A activity is upregulated in tolerized peritoneal macrophages, corresponding to decreased TNF-α secretion upon second LPS stimulation. We further examined the role of PP2A in the tolerance effect by using PP2ACα^lox/lox^;lyM-Cre conditional knockout macrophages. We found that PP2A phosphatase activity cannot be further increased by tolerance. TNF-α secretion from tolerized PP2ACα^lox/lox^;lyM-Cre macrophages is higher than tolerized control macrophages. Furthermore, we showed that the increased TNF-α secretion may be due to an epigenetic transcriptionally active signature on the promoter of TNF-α gene rather than regulation of the NFκB/IκB signaling pathway. These results suggest a role for increased PP2A activity in the regulation of immune tolerance.

## 1. Introduction

Protein phosphorylation plays a key role in the regulation of many cellular functions such as cell growth and differentiation, cell death, immune responses and neurological activities [1,2,3,4]. A balance between protein kinases and protein phosphatases regulates these phosphorylation-dependent responses. In addition to protein tyrosine (Tyr) phosphatases (PTPs), the protein Ser/Thr phosphatases (PSPs) compose another large protein phosphatase family. PSPs are classified into three subtypes: phosphoprotein phosphatases (PPPs), metal-dependent protein phosphatases (PPMs), and the aspartate-based phosphatases represented by FCP/SCP [5]. The PPP family is comprised of serine/threonine phosphatases including the most abundant protein phosphatases: PP1, PP2A and PP2B, as well as less abundant PP4, PP5, PP6 and PP7.

PP2A is a key cellular serine-threonine phosphatase, accounting for ~1% of total cell protein content. It is an endogenously active protein phosphatase whose activity is increased by NiCl_2_ [6]. PP2A exists in two forms—a heterodimeric core enzyme and a heterotrimeric holoenzyme. The PP2A core enzyme consists of a scaffold subunit (also known as the A or PR65 subunit) and a catalytic subunit (C subunit). The PP2A core enzyme interacts with variable regulatory subunits to assemble into more than 75 distinct PP2A holoenzymes. Although highly conserved within the same family, these regulatory subunits share little sequence similarity across families, and their expression levels vary greatly in different cell types and tissues [5].

For many immune cells, particularly macrophages, prior and extended exposure to LPS (and other Toll receptor agonists) renders the cells refractory or “tolerant” to subsequent endotoxin challenge [7] often characterized by attenuated TNF-α, IL-12 and KC production [8]. Previously, it has been suggested that various molecules, such as SHIP, IL-10, TGF-β, IRAK-M and MKP-1, were involved in this biologic response [7]. However, due to a lack of PP2A-specific phosphatase assay as well as non-specific inhibitors, the role of PP2A in endotoxin and/or cross-tolerance was not well studied. Herein, we utilized and optimized a fluorescence-based and immunoprecipitation-aided method to specifically measure and consequently investigate phosphatase activity of PP2A in tolerized macrophages. We found that tolerance significantly induced PP2A activity. We then used myeloid-specific, PP2ACα knockout macrophages (PP2ACα^l^°^x/l^°^x^;lyM-Cre) to further examine the precise regulatory role of PP2A on tolerance and TNF-α gene expression at both the cell signaling-transcriptional regulation level and chromatin modification level (epigenetic regulation).

## 2. Materials and Methods

### 2.1. Reagents

EnzCheck Ser/Thr phosphatase assay kit was purchased from molecular probes (Eugene, OR, USA). Pierce crosslink Magnetic IP/Co-IP kit was purchased from Thermo Scientific (Waltham, MA, USA). The chromatin immunoprecipitation (ChIP) assay kit (LowCell# ChIP) was purchased from Diagenode (Denville, NJ, USA). Difco thioglycollate medium was obtained from Becton, Dickinson and Company (Sparks, MD, USA). Anti-PP2A (C subunit, clone 1D6), normal mouse IgG were purchased from Upstate (Temecula, CA, USA). Phosphorylated antibodies (p-IKKα/β, p-IκBα, p-NFκB p65) and non-phosphorylated anti-IκBα were purchased from Cell Signaling (Danvers, MA, USA) respectively. Chip-grade H3K4me3 polyclonal antibody was purchased from Diagenode. LPS (*Escherichia coli* 055:B5) were purchased from Sigma-Aldrich (St. Louis, MO, USA). Mouse TNF-α Elisa kit was purchased from Invitrogen (Grand Island, NY, USA). Taqman primer/probe for PP2ACα was purchased from Applied Biosystems (Foster City, CA, USA).

### 2.2. Animals

All animal experiments were performed in accordance with the National Institutes of Health guidelines and locally approved by the University of Michigan’s Committee on Use and Care of Animals (UCUCA). Five to six weeks pathogen free C57BL/C mice were purchased from Charles River (Portage, MI, USA). Myeloid-specific PP2ACα conditional knockout mice (PP2ACα^l^°^x/l^°^x^;lyM-Cre) were established in the lab.

### 2.3. Peritoneal Macrophage Isolation and *in Vitro* Stimulation

Mice were injected intraperitoneally (i.p.) with 3% thioglycollate for four days. At day 4, peritoneal macrophages were harvested by repetitive (X3) lavaging of the peritoneal cavity via an eighteen-gauge needle attached to a 10 mL syringe. With the injection of 10ml of DPBS buffer to the cavity each time, ~30 mL of peritoneal fluid was obtained and cells were pelleted by centrifugation at 1200 rpm for 10 min. Cells were then re-suspended in RPMI 1640 medium (Invitrogen) containing 2% FBS (*Research Grade*, Fisher; Waltham, MA, USA) and plated into tissue culture plates. Macrophages were allowed to adhere for 2 h, and nonadherent ones were removed with gentle washing with warmed media before the final, fresh culture medium was added.

### 2.4. Phosphatase Assay

The cells were treated with LPS (100 ng/mL) for varying times at 37 °C in a humidified CO_2_ incubator (5% CO_2_ and 95% air). After designated incubation times, cells were washed and lysed with imidazole buffer (20 mM imidazole-HCl, 2 mM EDTA, pH 7.0, with protease inhibitors) for phosphatase assay. Cell lysates harvested from macrophages were measured for protein levels by BCA method (Pierce). Various amounts of proteins in 100 µL of 1 x reaction buffer containing 1 mM NiCl_2_ were added to a 96-well plate containing reconstituted Serine/Threonine phosphatase substrate-6,9-difluoro-4-methyl-umbellifery (DiFMUP). The substrate generates DiFMU after removal of the phospho-group, which exhibits fluorescence at 358/452 nM. After incubation at 30 °C for different time periods, plates were subjected to fluorescence reading on SpectraMax M3 plate reader (Molecular Devices, Sunnyvale, CA, USA). Wells devoid of either lysate or enzyme served as background measurements. Phosphatase activities were calculated by either fluorescence readings or by the absolute DiFMU amount present in the solutions based on the standard curve analysis.

### 2.5. Immunoprecipitation

Normal mouse IgG or anti-PP2A was incubated with Pierce Protein A/G Magnetic beads at room temperature for 15–30 min and then the beads were washed twice to remove unbound antibodies. Crosslinker Disuccinmidyl Suberate (DSS, 0.25 mM) was added to the antibody-bound beads and mixed on a rotator for 10–15 min and then quenched by the elution buffer provided by the Crosslink Magnetic IP/Co-IP kit. Cell lysates were incubated with antibody-crosslinked beads at room temperature for 1–2 h. After washing three times with washing buffer, the beads were incubated with 1× phosphatase reaction buffer containing reconstituted DiFMUP substrate and NiCl_2_. After incubation at 30 °C for 30 min, beads were removed by magnet and supernatants were measured for phosphatase activity.

### 2.6. Western Blot

Proteins harvested from macrophages were boiled in SDS sample buffer and subjected to 4%–12% NuPAGE (Invitrogen) gel electrophoresis followed by immunoblotting with anti-phospho-IKKα/β, anti-phospho-NFκB p65, anti-phospho-IκB-α antibody or IκB-α (1:1000) at 4 °C for overnight. Blot was detected by enhanced chemiluminescence (ECL prime, Amersham Pharmacia Biotech, Pittsburgh, PA, USA).

### 2.7. Chromatin Immunoprecipitation Assay (ChIP)

Peritoneal macrophages were isolated as described above. The chromatin immunoprecipitation (ChIP) procedure was performed by using LowCell# ChIP kit according to the manufacturer’s instructions. Briefly, 1 × 10^6^ cells were treated with or without 100 ng/mL LPS for 24 h. DNA-protein structure was cross-linked by 1% formaldehyde for 10 min at room temperature. Cells were collected and lysed with 130 µL SDS lysis buffer and resulting lysates were sonicated to obtain DNA fragments ranging from 200 to 1000 bp (base pairs) using a Diagenode Bioruptor for three runs of five cycles (30 s on, 30 s off). After centrifugation, chromatin DNA was diluted, and an aliquot (10% volume) was saved to indicate the input DNA in each sample. The remaining chromatin fractions were incubated with anti-H3K4me3-coated magnetic beads overnight at 4 °C with gentle rotation. After washing, the beads were incubated with Proteinase K at 55 °C for 15 min, followed by inactivation at 100 °C for 15 min. DNA remaining in the supernatant was purified by standard phenol/chloroform and ethanol precipitation and was subjected to real-time PCR. Primers for mTNF-α promoter are as follows: forward, GGAAATAGACACAGGCATGGTCT; reverse, CCTACACCTCTGTCTCGGTTTCTT. Data was expressed as percentage of starting material: % (ChIP/Total input).

### 2.8. Statistical Analysis

All statistics were performed using Graphpad Prism 4 (Graphpad, San Diego, CA, USA). Values were expressed as the mean ± standard error of the mean. Significance was assigned for *p* < 0.05. Data sets were analyzed using student’s *t*-test or one-way analysis of variance (ANOVA), with individual group means being compared with the Student-Newman-Keuls post-test.

## 3. Results

### 3.1. Fluorescence-Based Measurement of Phosphatase Activity Changes in LPS-Stimulated Peritoneal Macrophages

**Figure 1:**
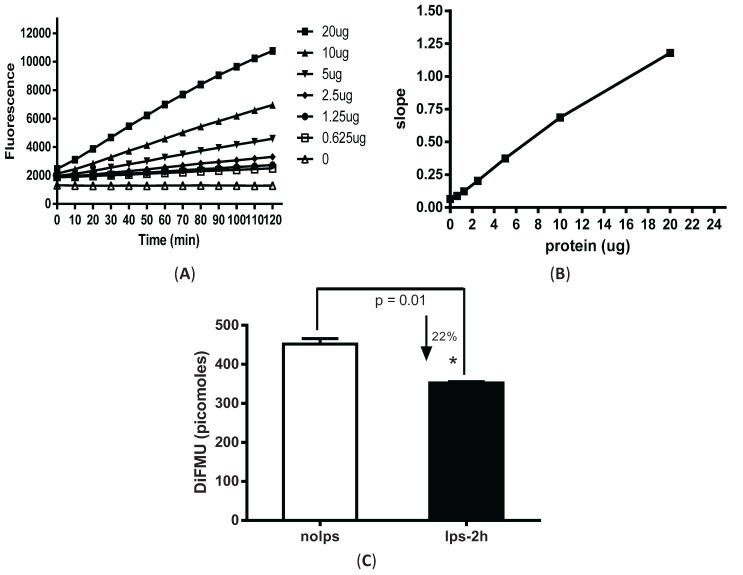
Fluorescence-based phosphatase assay in LPS-stimulated peritoneal macrophages. Elicited peritoneal macrophages were harvested from C57BL6 mice at day 4 after thioglycollate injection. Cells were lysed with imidazole buffer and protein concentration was measured by BCA assay. (**A**) Time-course and dose-dependent responses measured from total cell lysates in buffers containing non-fluorescent substrate DiFMUP and NiCl_2_ additive. Y-axis shows the fluorescence reading as random light units; (**B**) Slope reaction. Slope value represents reaction rates which are calculated by dividing the changes of enzyme activity by time (from kinetic reaction). Assay kinetic was set from 0 to 120 min with an interval value of 10 min; (**C**) Down-regulation of phosphatase activity in LPS-stimulated macrophages (100 ng/mL LPS for 2 h). Absolute value of DiFMU on the Y-axis was calculated based on DiFMU standard curve. * *p* = 0.01 as compared to non-LPS treated cells.

Elicited peritoneal macrophages were harvested from mice four days after intraperitoneal injection of 3% thioglycollate. An active phosphatase removes the phospho-group from DiFMUP and the generated fluorescent DiFMU can be measured at 358/452 nM on SpectraMax M3 plate reader. Varied amounts of total cellular protein (0.625–20 µg) were added to 96-well plate pre-loaded with DiFMUP (50 µM) in reaction buffer containing 1 mM NiCl_2_. The mixture was incubated at 30 °C for varied time points (0–120 min). With all the protein amounts tested (0.625–20 µg of cell lysates), linear enzymatic responses were achieved for the complete duration of the reaction (Figure 1A). Given that PP2A constitutes ~1% of total cellular protein, the estimated enzyme amounts used in the assay was ~6.25–200 ng. For consistency, 5 µg of cell lysate was used to measure PP2A activity in our subsequent experiments, unless otherwise specifically stated. Slopes were calculated by dividing the changes of enzyme activity by time (resulting from kinetic reaction) to represent the rate of the reaction. The slope value was plotted against the input protein amount (Figure 1B). Continuously increased slope values were observed with correspondingly increasing protein amounts that was consistent with the linear enzymatic reaction shown Figure 1A.

After the assay conditions were optimized for peritoneal macrophages, we examined the changes in phosphatase activity from cells stimulated with LPS. Macrophages stimulated with LPS for only 2 h showed a significant decrease (~22%) in phosphatase activity (Figure 1C).

### 3.2. LPS Pre-Treatment Induced Tolerization and Upregulation of Phosphatase Activity in Peritoneal Macrophages

**Figure 2:**
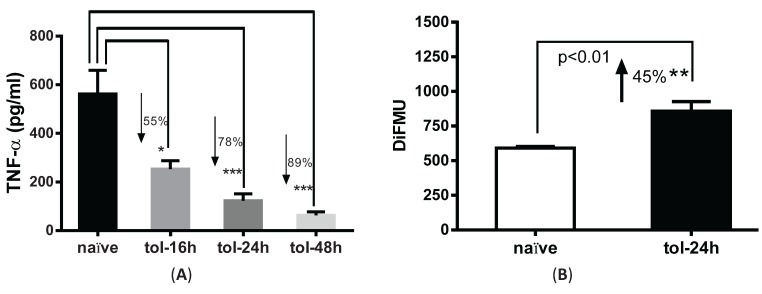
Pre-treatment with LPS induced tolerance and upregulation of phosphatase activity in peritoneal macrophages. (**A**) Peritoneal macrophages harvested from C57BL6 mice were pre-treated with 100 ng/mL LPS for 16, 24 and 48 h and subjected to second LPS stimulation for 4 h. Supernatants were collected and measured for TNF-α secretion. * *p* < 0.05; *** *p* < 0.001 as compared to naïve cells; (**B**) Phosphatase activity in LPS-tolerized macrophages. Cells were treated with 100 ng/mL of LPS for 24 h, lysed and subjected for phosphatase assay. Y axis shows the absolute value of DiFMU present in the solution. ** *p* < 0.05 as compared to non-tolerized cells.

We next used this fluorescence method to examine phosphatase activity in LPS-tolerized cells. To test the tolerance effect, peritoneal macrophages were pre-treated with or without 100 ng/mL LPS for 16, 24 and 48 h and re-stimulated with 100 ng/mL LPS for 4 hrs. Consistent with prior reports [9], TNF-α secretion was decreased by 55%, 78% and 89% in the LPS-pretreated cells *versus* naïve cells at the indicated time points (Figure 2A). For consistency, we used 24 h time point as the tolerant condition in the remaining studies. Phosphatase activity in tolerized macrophages was significantly increased by ~45% (*p* < 0.01) after cells were tolerized for 24 h as compared to naïve cells (Figure 2B).

### 3.3. Immunoprecipitation-Aided Fluorescence Method to Examine PP2A Activity in Tolerized Macrophages

**Figure 3:**
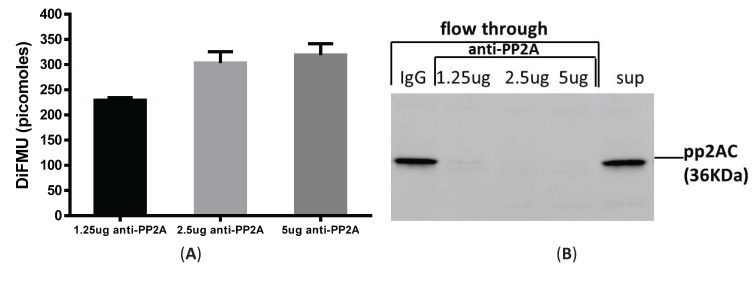

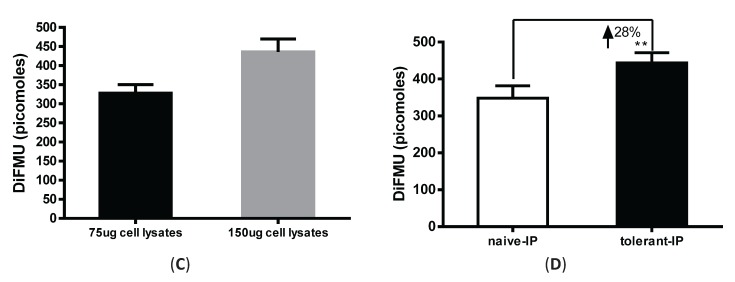
Immunoprecipitation-aided fluorescence method of measuring PP2A activity in tolerized peritoneal macrophages. Anti-PP2A IgG was incubated with Protein A/G magnetic beads for 30 min and then cross-linked to the beads by DSS. Cell lysates were then incubated with antibody-conjugated beads for 1 hr at room temperature and the beads were removed of were washed to remove non-specific binding. Assay buffer containing DiFMUP and NiCl_2_ was added to the beads, mixed and incubated at room temperature for 30 min. Supernatant removed of beads was measured on a SpectraMax M3 plate reader. (**A**) Anti-PP2A dose-response. Three doses of anti-PP2A IgG were cross-linked to the beads and incubated with the same amount of input lysates (75 µg) for the phosphatase assay; (**B**) Flow through section after beads were removed was examined for the presence of PP2A to determine pulldown efficiency; (**C**) Input cell lysates dose-response. Same amount of anti-PP2A IgG (2.5 µg) was incubated with two doses of cell lysates (75 µg and 150 µg). PP2A activity was measured by DiFMU method. Y axis shows the absolute values of DiFMU in solutions; (**D**) LPS tolerance induced upregulated PP2A-activity in peritoneal macrophages. 75 µg of cell lysates harvested from naïve or tolerant peritoneal macrophages were subjected to immunoprecipitation with 2.5 µg of anti-PP2AC antibody. Y-axis shows the absolute values of DiFMU.

It is argued that PP2A activity can be distinguished from PP1 and PP2B by adding different metal ions, such as NiCl_2,_ MnCl_2_ and Ca^2+^ to the assay buffer, which can differentially enable phosphatase activities [10]. However, other low abundant phosphatases in the PPP family (e.g., PP4, PP5 and PP6) are known to interfere with the assay specificity [11]. To measure PP2A-specific activity in LPS-tolerized cells, we combined immunoprecipation strategy to pull down PP2A from total cell lysates and then examined the phosphatase activity from these precipitates using the fluorescence assay. We optimized the antibody amount and cell lysate amounts by performing a titration experiment using various concentrations of anti-PP2A antibody and cell lysates. As shown in Figure 3A, 75 µg of lysates from elicited peritoneal macrophages was incubated with 1.25 μg, 2.5 μg and 5 μg of anti-PP2A antibody cross-linked to Protein A/G magnetic beads (see “Materials and Methods”). Increased phosphatase activity was readily detected from 1.25 μg to 2.5 μg of anti-PP2A, with no further increase detected using 5 μg anti-PP2A. To determine the pulldown efficiency of PP2A from the lysates, flow through eluent after beads were removed was examined for PP2A by western blot. As shown in Figure 3B, no PP2A protein was detected in the flow through segment with 2.5 µg and 5 µg anti-PP2A pull down antibody. The beads were also blotted with anti-PP2A and anti-PP1 antibody to examine the specificity of this pulldown experiment. While PP2A is ready detected on the beads, no signal for PP1 was detected on the beads based on antibody specificity [12]. Therefore, we fixed the anti-PP2A antibody amount at 2.5 µg, and compared phosphatase activities with two sample concentrations (75 and 150 μg cell lysate). A concentration-dependent increase in phosphatase activity was clearly detected (Figure 3C). Therefore, 2.5 μg of anti-PP2A antibody and 75 µg of cell lysates were used thereafter to examine the PP2A activity under the various experimental conditions. Consistent with Figure 2B, an increase of PP2A activity was observed in tolerized macrophages by this immunoprecipitation method, albeit to lesser degree (~28% increase in Figure 3D compared to ~45% increase in Figure 2B). However, this change likely reflects PP2A-specific upregulation, suggesting that PP2A plays a regulatory role in tolerance. In the whole cell lysates assay method, PP2A activity is measured by adding ZnCl_2_ to the assay buffer, which cannot completely exclude the activities from other phosphatases in the lysates.

### 3.4. PP2ACα-Conditional Knockout Macrophages Released More TNF-α upon LPS Stimulation in both Naïve and Tolerized Cells

**Figure 4:**
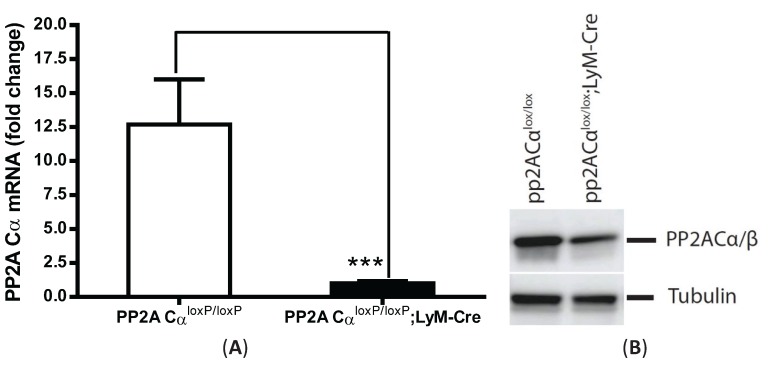

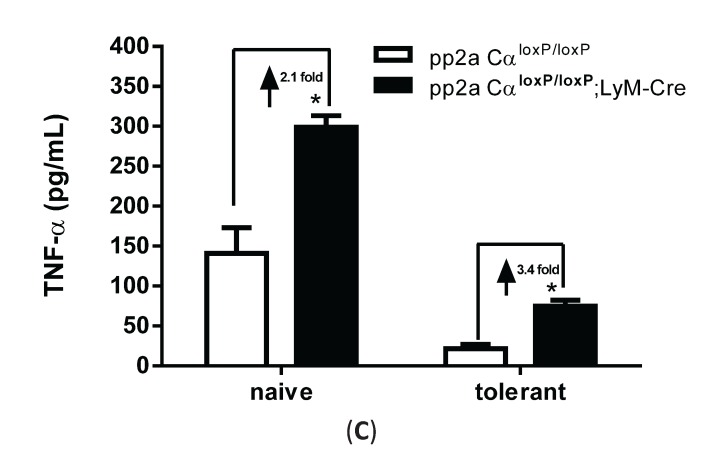
Increased TNF-α secretion from PP2ACα conditional knockout macrophages under both naïve and tolerant conditions. Elicited peritoneal macrophages were isolated from control PP2ACα^l^°^x/l^°^x^ mice and PP2ACα^l^°^x/l^°^x^;LyZ-Cre mice. (**A**) Q-PCR analysis of PP2ACα mRNA expression. *** *p* < 0.001 as compared to control macrophages; (**B**) Western blotting for the catalytic subunit of PP2A (α and β isoforms) in control and PP2ACα knockout macrophages; (**C**) TNF-α secretion from naïve and tolerized cells. Both control and PP2ACα knockout cells were pre-treated with (tolerized) or without (naïve) 100 ng/mL LPS for 24 h and then re-stimulated with second LPS for 4 hrs. Supernatants were measured for TNF-α secretion by ELISA. * *p*< 0.05 as compared to naïve cells.

To further investigate whether upregulation of PP2A activity played a role in tolerized macrophages, we compared TNF-α secretion from macrophages isolated from pp2aCα^l^°^x/l^°^x^ mice in the absence of any Cre (“control mice”) as compared to that from myeloid-specific PP2ACα conditional knockout mice. PP2ACα^l^°^x/l^°^x^;lyM-Cre mice were established by breeding PP2ACα^l^°^x/l^°^x^ mice with lyM-Cre mice to obtain myeloid-specific gene knockout of PP2ACα [13]. We verified conditional knockdown of PP2ACα using quantitative-PCR with PP2ACα-specific primers to show significantly diminished PP2ACα mRNA expression (Figure 4A). This conditional loss of transcriptional expression resulted in significantly decreased PP2A protein expression as measured by western blot analysis using an antibody against epitope 295–309 amino acids of the catalytic subunit of PP2A (Figure 4B). This smaller reduction in apparent PP2AC protein as compared to the significant reduction in PP2ACα mRNA is likely due to the presence of the PP2ACβ isoform in the cells as the anti-PP2AC antibody we used cannot distinguish these two isoforms [14]; currently there is no α-isoform specific anti-PP2A antibody. These two isoforms are encoded by genes at different chromosome locations but share 97% identical residues though PP2ACα transcript are about 10-fold more abundant than the PP2ACβ transcript.

Employing the endotoxin tolerance model, 24 h after pretreatment with LPS, cells were washed (X 2) with new medium and re-stimulated with 100 ng/mL LPS for 4 h. In naïve cells, the amount of TNF-α secretion from knockout cells was increased by 2.1-fold compared to control macrophages (Figure 4C). In tolerized cells, both cells showed a tolerized phenotype as shown by an 82% decrease of TNF-α secretion in tolerized control cells and a 75% decrease in tolerized knockout cells. However, the total amount of secreted TNF-α from tolerized PP2ACα^l^°^x/l^°^x^;lyM-Cre remained higher (increased by 3.4-fold) than tolerized PP2ACα^l^°^x/l^°^x^ macrophages.

### 3.5. LPS Tolerance Did Not Change PP2A Activities in PP2ACα^l^°^x/l^°^x^;lyM-Cre Conditional Knockout Macrophages

**Figure 5:**
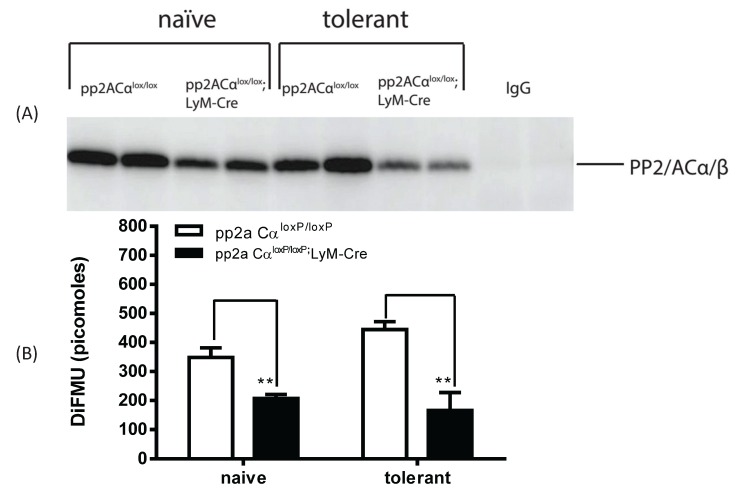
PP2A activity in control and pp2ACα knockout macrophages under both naïve and knockout conditions. Elicited peritoneal macrophages were pretreated with (tolerant) or without (naïve) 100 ng/mL LPS for 24 h. Total cell lysates were incubated with antibody cross-linked beads (control mouse IgG or anti-PP2A IgG) as described in Figure 3. Beads were incubated with phosphatase assay buffer containing DiFMUP and NiCl_2_ at 30 °C for 30 min. Supernatants were then removed and assayed for phosphatase activity. (**A**) Beads were boiled in 2 × SDS-PAGE loading buffer and protein bound to the beads was probed for PP2A; (**B**) PP2A activity. In control cells (PP2ACα^l^°^x/l^°^x^), tolerance induced an upregulation of phosphatase activity; whereas in PP2ACα knockout macrophages (PP2ACα^l^°^x/l^°^x^;lyM-Cre), PP2A activity was unchanged.

We used the immunoprecipitation and phosphatase assay conditions established above (see Figure 3) to measure PP2A activity in naïve and tolerized macrophages isolated from either control (pp2aCα^l^°^x/l^°^x^) or PP2ACα knockout mice (pp2aCα^l^°^x/l^°^x^;lyM-Cre). Western blot analysis showed that the amount of PP2A protein bound to the beads was significantly reduced for the knockout macrophages as compared to those from the pp2aCα^l^°^x/l^°^x^ mice (Figure 5A). (Note, no signal was detected on beads cross-linked with control IgG). Using 75 µg of total lysates and 2.5 µg of anti-PP2A antibody we measured PP2A activity in the cells. Consistent with a lesser amount of PP2A bound to the beads, a ~50% reduction in phosphatase was detected in pp2aCα^l^°^x/l^°^x^;lyM-Cre macrophages in comparison to control cells under both naïve and tolerant conditions (Figure 5B). However, in contrast to control cells, tolerant conditions did not induce changes of PP2A activity in PP2ACα knockout cells (Figure 5B) which may be related to the inability to transcriptionally drive PP2A production.

### 3.6. PP2ACα Deletion Did Not Induce Phosphorylation-Mediated Degradation of IκBα

**Figure 6:**
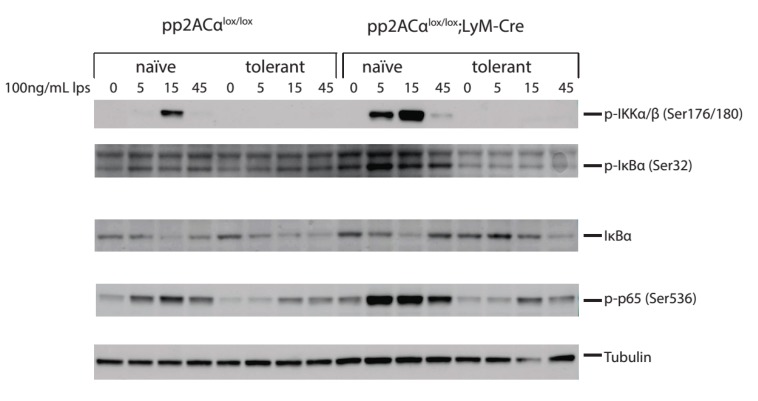
Effects of LPS tolerance and PP2ACα knockout on NFκB/IκB signaling. Elicited peritoneal macrophages were isolated from control PP2ACα^l^°^x/l^°^x^ mice and PP2ACα^l^°^x/l^°^x^;lyM-Cre mice. Both cells were pre-treated with (tolerant) or without (naïve) 100 ng/mL LPS for 24 h. Cells were then washed and re-stimulated with second LPS for 0, 5, 15 and 45 min. Cell lysates were subjected to protein gel electrophoresis and western blot analysis with phospho-protein specific antibodies and endogenous control antibody.

We next investigated the molecular mechanisms of PP2ACα gene deletion-mediated increase of TNF-α secretion in both naïve and tolerized cells. TNF-α gene transcription is primarily regulated by the NF-кB pathway [15]. At the promoter region of TNF-α, three NF-κB binding sites are specifically recognized by NF-κB to initiate transcription [16]. LPS stimulation induced signaling cascades activate IKK (IκB Kinase) complex [17]. IKK phosphorylates the inhibitor of NF-κB, IκBα on the Serine 32 and 36 residues that targets it for ubiquitination-mediated degradation [18]. IκBα degradation releases NF-κB from the inhibitory complex. Released NF-κB proteins are transported into the nucleus where they bind to their target sequences to activate gene transcription [19]. We therefore performed western blot analyses to probe the key mediators of NF-κB pathway (IKKα/β, NFκB-p65 and IκBα) with phospho-specific antibodies. In naïve cells, PP2ACα knockout caused significantly increased phosphorylation on IKKα/β, IκBα and NFκB-p65 at 5 and 15 min (Figure 6). This upregulation is consistent with the reports that PP2ACα binds directly to and dephosphorylates phospho-IKKα/β and phospho-NFκB p65 [20,21]. Notably, although phosphorylation of IκBα in PP2ACα knockout cells was increased, it did not enhance degradation of non-phosphorylated IκBα. In tolerized cells, the phosphorylation state of IKKα/β, IκBα and NFκB p65 was substantially reduced in both control and PP2ACα knockout macrophages (Figure 6), whereas non-phosphorylated IκBα was unchanged. These results indicated that transcriptional activation via IκBα degradation may not be responsible for the increased TNF-α secretion resulting from gene deletion of PP2ACα.

### 3.7. PP2ACα Knockout Induced Increased Binding of H3K4me3 to the Promoter Region of TNF-α after LPS Tolerance

**Figure 7:**
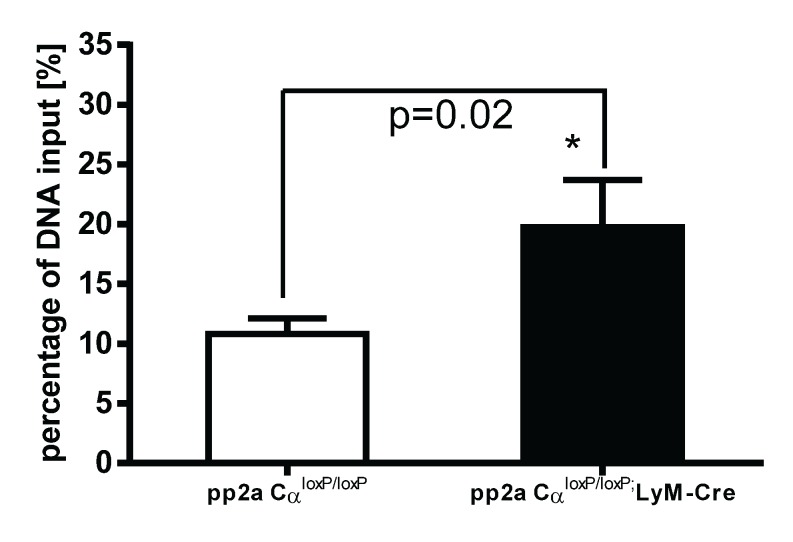
Epigenetic regulation of TNF-α gene expression in tolerized control and PP2ACα knockout macrophages. Elicited peritoneal macrophages were isolated from control PP2ACα^l^°^x/l^°^x^ mice and PP2ACα^l^°^x/l^°^x^;lyM-Cre mice. Both cells were pretreated with 100 ng/mL of LPS for 24 h and then washed and re-stimulated with second LPS (100 ng/mL) for 2 h. Chip-IP procedure was conducted as described in “Materials and Methods”. DNA samples subjected to immunoprecipitation were analyzed for the presence of TNF-α promoter sequences in the DNA/protein complex by Q-PCR and obtained CT values were standardized with the CT values from non-immuoprecipitated input DNA. Data was expressed as percentage of input DNA: % (ChIP/Total input). In these tolerized cells, PP2ACα knockout cells shows more binding of the epigenetic “on” signal (H3K4Me3) to the promoter region of TNF-α gene as compared to control cells (* *p* < 0.05).

In recent years, it was found that epigenetic modification of DNA sequences provides another level of regulation of gene expression [22]. Modification of histone proteins by acetylation, methylation or phosphorylation affects the degree of DNA winding and unwinding thus affecting transcriptional machinery accessibility or inaccessibility to drive gene expression. Examples include trimethylation of lysine 4 of histone 3 (H3K4me3) which generally enhances access to DNA leading to gene activation, whereas trimethylation of lysine 27 of histone 3 (H3K27me3) closes DNA resulting in a gene silencing effect [23,24]. To further understand the mechanisms of increased TNF-α secretion in tolerized PP2ACα knockout macrophages, we examined the epigenetic regulation on TNF-α gene expression. Both control (pp2aCα^l^°^x/l^°^x^) and knockout (pp2aCα^l^°^x/l^°^x^;lyM-Cre) peritoneal macrophages were tolerized with 100 ng/mL LPS for 24 h and then washed and re-stimulated with second LPS for 2 h. As shown in Figure 7, in PP2ACα knockout cells, binding of H3K4me3 to the promoter region of TNF-α was significantly increased compared to the binding in control macrophages. These data indicate that epigenetic regulation may play a role in the upregulation of TNF-α secretion from tolerized pp2aCα^l^°^x/l^°^x^;lyM-cre macrophages.

## 4. Discussion

PP2A is among the most highly expressed and constitutively activated intracellular phosphatases. In both monocytes and macrophages, high basal PP2A activation was reported [2,25]. The traditional phosphatase assay has used malachite green as a color indicator of released free phosphate, but it gives a low sample *versus* control ratio due to the binding of malachite green to ubiquitous free phosphate present in most cell lysates. Previously, we have used malachite green method to measure PP2A activity changes upon short time points of LPS stimulation. However, the changes are subtle and difficult to quantitate. In this study, we used a fluorescence-based assay which utilizes 6,8-difluoro-4-methylumbelliferyl phosphate (DIFMUP) as a substrate to avoid the concerns of free phosphate contamination [26]. With this new method, a ~22% steady decrease of phosphatase activity was detected from macrophages stimulated with LPS for 2 h. (Figure 1C). Furthermore, we found that in tolerized cells, significantly increased phosphatase activity (~45%) was observed (Figure 2B). However, since the assay relies on divalent cations (NiCl_2_ for PP2A, MnCl_2_ for PP1 and Ca^2+^ for PP2B) to differentiate among Ser/Thr phosphatases [27], the activity change may not be completely PP2A-specific. For example, PP1 and PP2A are both ubiquitously present in cells and share 67% homology [28]. Previously varied chemical and recombinant peptide inhibitors have been tried to distinguish these two phosphatases but results have demonstrated imprecise distinction [29,30]. Therefore, to rule out the possible contamination of other serine/threonine phosphatases in this fluorescent method, we modified this assay by immunoprecipitating PP2AC from the lysates using anti-PP2AC conjugated magnetic beads and then measuring phosphatase activity directly on the protein conjugated to the beads. PP2A-specificity of this assay was confirmed by immunoblotting both PP2A and PP1 on the beads. While PP2A was readily detected on the beads, no signal was shown for PP1 with anti-PP1 antibody [12]. With this PP2A-specific assay, we also detected significantly upregulated PP2A activities in LPS tolerized peritoneal macrophages, which suggest that PP2A plays roles in endotoxin tolerance. We therefore investigated PP2A regulation by examining the tolerance effects using PP2ACα conditional knockout macrophages (PP2ACα^l^°^x/l^°^x^;lyM-Cre). We confirmed that PP2ACα gene knockout significantly increased TNF-α secretion from naïve cells confirming a role for PP2A in regulating LPS induced TNF-α expression as we previously reported [25]. In tolerized cells, TNF-α secretion from both control and PP2ACα knockout macrophages were reduced, but TNF-α from PP2ACα knockout cells still remained significantly more than control cells (Figure 4C).

It is known that TLR4 engagement induces MyD88 recruitment to TLR4, which leads to IRAK kinase activation [31], followed by activation of mitogen activated protein (MAP) kinase pathways (e.g., p38, JNK, and ERK), and NF-κB pathway [32]. In tolerized cells, it was demonstrated that recruitment of MyD88 was impaired and IRAK activation became defective [33]. Similarly, MAP kinases [34], IκB kinase [35] and NF-κB [36] were deactivated, which was associated with reduced production of cytokines, such as TNF-α, IL-1β and IL-6, *etc.* [37]. Phosphatases have been shown to be negative regulators of signal transduction by their capability to dephosphorylate proteins that often results in their inactivation. For example, MKP-1, a primary member of the dual-specificity phosphatase that preferentially dephosphorylates activated p38 MAPK and c-Jun N-terminal Kinase (JNK), was demonstrated to play a role in endotoxin tolerance [38]. PP2A, on the other hand, has been demonstrated to interact with activated signaling proteins at multiple levels. For example, co-immunoprecipitation study identified associations of distinct PP2A core or holoenzymes with the IKK, NF-κB and TRAF2 complexes [21]. Moreover, direct interaction and dephosphorylation of RelA with PP2A was demonstrated in Sulindac treated melanoma cells [39]. Therefore, we surmised that PP2A was actively involved in tolerance effects by regulating these key kinases. TNF-α is the major proinflammatory cytokine secreted from LPS-stimulated macrophages and at gene transcription level, TNF-α expression is largely regulated by the NF-кB pathway. In the cytosol in resting cells, non-phosphorylated IκB-α associates with NF-κB and inhibits nuclear transloaction of this transcription factor. Upon stimulation, IκB-α is phosphorylated by activated IKKβ [18], ubiquitinated and undergoes proteasomal degradation releasing NF-κB to translocate to the nuclear compartment to activate gene transcription [40]. However, in this study, we found that although gene deletion of PP2ACα in macrophages substantially increased the phosphorylation of IKKα/β, NFкB-p65 and IкBα molecules in naïve cells, the stability of non-phospho-IкBα protein was unchanged (Figure 6). Furthermore, we found that tolerance causes deactivation of NFκB signaling in both control and PP2ACα deletion cells without changing the stability of IкBα protein as well (Figure 6). Taken together, the data lead us to investigate other levels of gene regulation which might be responsible for the elevated TNF-α secretion in the PP2ACα knockout cells after tolerance.

We now understand that gene expression is not only dictated by the genetic code, but also by epigenetic alterations of chromatin as histone proteins regulate gene transcription by binding to the promoter regions of genes. Post-translational modification (methylation, acetylation, *etc.*) on histone proteins can change the accessibility of the promoter regions to transcription factors and other regulatory proteins, which leads to either an activation or deactivation of gene transcription. In this study, we performed Chip-IP assay to evaluate the binding of TNF-α promoter to the ‘gene on’ histone signature, H3K4me3, in tolerized macrophages. As compared to control cells, we found significantly increased binding of H3K4me3 to the promoter region of TNF-α gene in PP2ACα knockout cells as compared to control cells. Although other possible regulatory mechanisms, such as post-transcriptional regulation on TNF-α mRNA stability or post-translational regulation on TNF-α protein might also be responsible for the upregulated TNF-α secretion in tolerized PP2ACα knockout cells [25], our data indicate that PP2A is a negative regulator affecting histone modifications and consequently epigenetic regulation might play an important role in attenuating TNF-α gene expression during tolerance.

A potential epigenetic regulatory role of PP2A has been suggested in prior reports. For example, in systemic lupus erythematosus (SLE) model, it was found that PP2Ac transgenic mice produced high levels of IL-17 production from CD4 T cells after induction of glomerular injury [41] and this increased production was later on found to be due to enhanced histone 3 acetylation on the IL-17 locus associated with PP2Ac overexpression [42]. Another example of epigenetic regulation by PP2A was found in an acute pancreatitis model, where de-phosphorylation of class II HDACs by PP2A triggered nuclear localization of histone deacetylases, which eventually lead to increased gene repression [43]. Finally, several investigators have examined the biology of tolerant or reprogrammed cells because of the potential clinical relevance to immunoparalysis reported in the late phase of sepsis. In the acute phases of sepsis, a dysregulated inflammatory response mediated by innate immune cells stimulated by microbial infection results in an early overwhelming “cytokine storm.” This phase is often followed by a counter-regulatory, anti-inflammatory response thought to mediate the later “immune paralyzed” stage [44]. In mice subjected to a survivable model of sepsis (e.g., cecum ligation and puncture [CLP] model), isolated alveolar macrophages show decreased phagocytic activity and attenuated proinflammatory cytokine production [8]. It was demonstrated that impaired TNF-α production was associated with the reduced methylation of H3K4me3 to the promoter region of these genes [45], however, whether this is related to PP2A activation as suggested by the current data has not been explored.

## 5. Conclusions

By using fluorescence-based and immunoprecipitation-aided methodology, we demonstrated that PP2A plays roles in lps-induced immune tolerance. Furthermore, our study showed that PP2A regulates the tolerant effects (*i.e.*, attenuated TNF-α secretion from tolerized macrophages) by an epigenetic mechanism.
